# Proton-density fat-saturated non-contrast MR imaging is comparable to post-contrast MR imaging for revealing wrist synovitis

**DOI:** 10.1186/s13244-026-02406-1

**Published:** 2026-10-09

**Authors:** Su Wu, James Francis Griffith, João Dourado, Lai Shan Tam

**Affiliations:** 1 Department of Imaging and Interventional Radiology, The Chinese University of Hong Kong, Hong Kong, China; 2 Department of Radiology, Hospital de São João, Unidade Local de Saúde São João, Porto, Portugal; 3 Department of Medicine & Therapeutics, The Chinese University of Hong Kong, Hong Kong, China

**Keywords:** Rheumatoid arthritis, Synovitis, Contrast enhancement, MRI

## Abstract

**Objectives:**

Standard MR imaging (MRI) for rheumatoid arthritis (RA) patients comprises a post-contrast T1-fat-saturated (T1FS + C) sequence to detect wrist synovitis and tenosynovitis. This study investigates whether non-contrast sequences can provide similar information to post-contrast imaging.

**Materials and methods:**

Seventy-seven RA patients (F/M:  64/13; age: 61 ± 12 years) underwent MRI examination of the most symptomatic wrist on a 3.0-T system. Axial T1FS + C as well as proton-density imaging with fat-saturation (PDFS) and T2-weighted imaging with fat-saturation (T2FS) sequences were performed. Two radiologists independently scored synovitis and tenosynovitis severity on all sequences, using the rheumatoid arthritis magnetic resonance imaging scoring system (RAMRIS). Intra- and interclass correlation coefficients (ICCs) were assessed.

**Results:**

For synovitis severity scoring, PDFS was practically identical to T1FS + C, while T2FS overestimated synovitis severity by up to 19%. For synovitis severity, PDFS imaging correlated better with T1FS + C than T2FS imaging (ICC: Reader 1: 0.931 vs 0.897; Reader 2: 0.969 vs 0.948). For tenosynovitis severity, PDFS and T2FS both yielded higher tenosynovitis scores than T1FS + C (*p* < 0.001). Compared to T1FS + C imaging, PDFS overestimated tenosynovitis severity by up to 39%, while T2FS overestimated tenosynovitis severity by up to 54%. For tenosynovitis severity, PDFS imaging correlated better with T1FS + C than T2FS imaging (ICC: Reader 1: 0.856 vs 0.731; Reader 2: 0.808 vs 0.800).

**Conclusion:**

PDFS imaging had good (for tenosynovitis) to excellent (for synovitis) intra- and inter-reader reliability and provides comparable scoring to T1FS + C imaging when evaluating synovitis severity, though it tends to overestimate tenosynovitis severity. PDFS imaging can reasonably replace contrast-enhanced imaging for assessing wrist synovitis in RA patients.

**Key Points:**

***Question*** Post-contrast imaging is recommended for evaluating synovitis and tenosynovitis in rheumatoid arthritis (RA) patients, though it involves higher cost, scan time, and discomfort.

***Findings*** Proton-density imaging with fat-saturation (PDFS) imaging had good to excellent intra- and inter-reader reliability for evaluating synovitis and tenosynovitis in RA wrists. Compared to post-contrast T1-fat-saturated (T1FS + C) imaging, PDFS imaging provides comparable semi-quantitative synovitis severity grading but tends to overestimate tenosynovitis severity.

***Critical relevance statement*** Post-contrast imaging for synovitis and tenosynovitis in RA patients involves discomfort, a longer time, and cost. PDFS imaging has good reliability and compares well with post-contrast imaging. In RA wrists, compared to T1FS+C imaging, PDFS imaging provides comparative semi-quantitative synovitis severity grading but tends to overestimate tenosynovitis severity.

**Graphical Abstract:**

**Figure:**
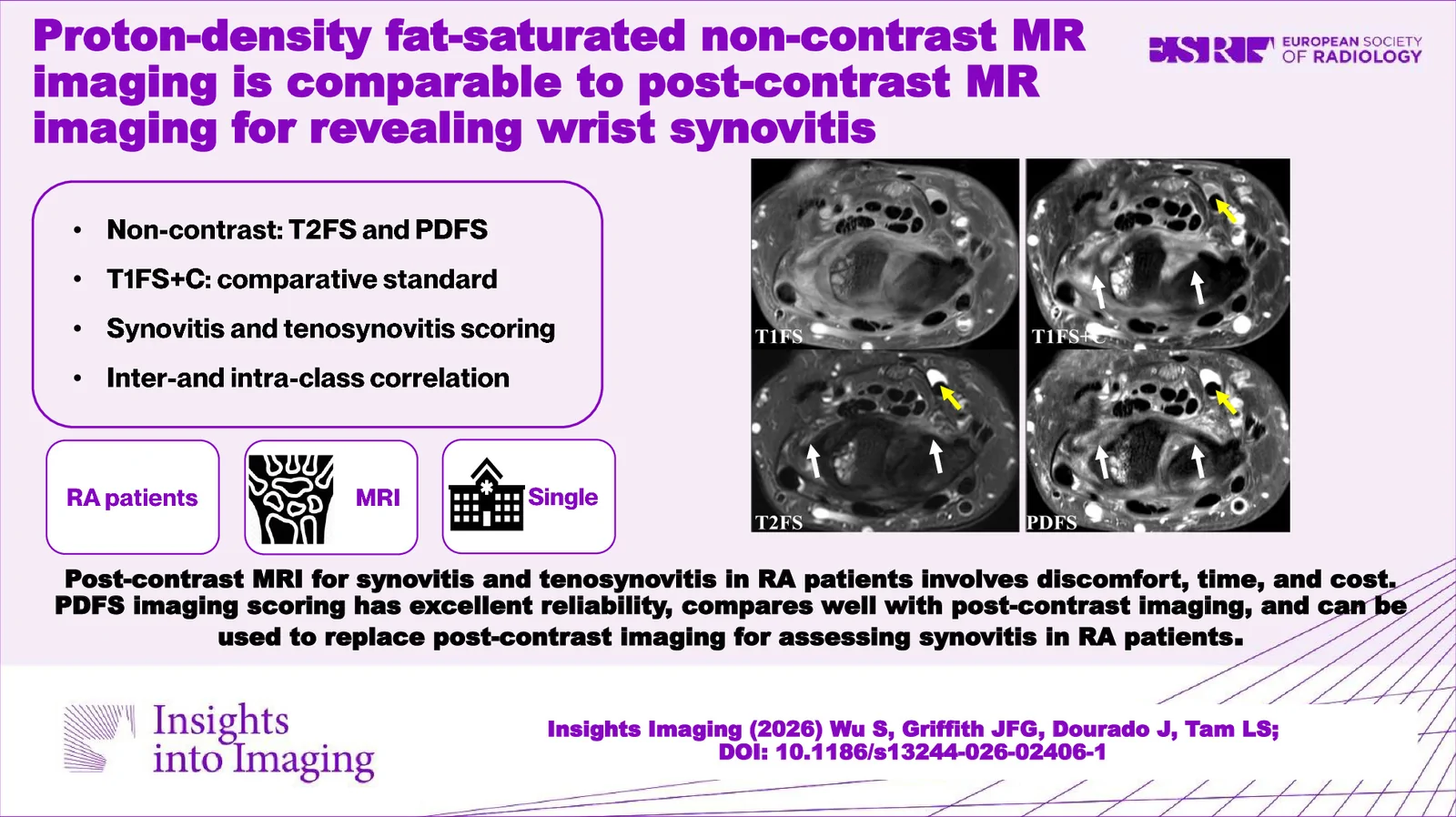

## Introduction

In rheumatoid arthritis (RA) patients, MR imaging (MRI) exquisitely reveals all clinically relevant markers of bone and soft tissue inflammation, namely bone marrow edema, synovitis, and tenosynovitis [1, 2]. The severity of ongoing inflammation is the most important parameter to consider when determining the need for treatment or treatment-change in RA patients [3]. The severity of synovitis present is more relevant than the presence of synovitis, as most RA patients will have some degree of residual synovitis even when in clinical remission [4–7]. Ultrasound is very helpful at detecting and grading the severity of synovitis and tenosynovitis in RA patients [8, 9]. Ultrasound, however, cannot quantify the degree of synovitis as efficiently as MRI, is not as sensitive at detecting erosions or joint space narrowing, and cannot identify bone marrow edema [8, 10].

A standard MR scan protocol for RA patients includes T1-weighted fat-saturated imaging after contrast to detect and quantify synovitis and tenosynovitis [4–7]. Synovitis seen on MRI correlates well with synovitis seen histologically [11]. The Rheumatoid Arthritis Magnetic Resonance Imaging Score (RAMRIS) system is a widely applied method for semi-quantifying synovitis, tenosynovitis, and bone marrow edema in RA patients [7, 12]. For evaluating bone marrow edema (‘osteitis’) and structural damage (bone erosion, joint space narrowing), non-contrast imaging is preferred [12–14].

Administering intravenous gadolinium contrast has several disadvantages. It requires venous cannulation, which is time-consuming, requires expertise, and is uncomfortable for patients. There is also a low risk of adverse effects to contrast agent such as acute kidney injury, nephrogenic systemic fibrosis, as well as mounting patient and medical concerns related to gadolinium retention in brain tissues, prompting prudence, whenever possible, on the use of gadolinium contrast [15]. Gadolinium is also costly, and post-contrast imaging prolongs examination time. That said, gadolinium administration is undoubtedly helpful at depicting tissue perfusion and is the current standard for identifying synovial inflammation. Nevertheless, it is feasible that non-contrast sequences could provide similar information without the need for gadolinium injection.

This study was designed to investigate whether other sequences could also accurately quantify soft tissue inflammation in the wrists of RA patients. Two frequently used sequences, namely axial T2WI fat-saturated (T2FS) and axial PDWI fat-saturated (PDFS), were added to the routine MRI wrist protocol for RA patient assessment. Axial T1WI fat-saturated post-contrast images (T1FS + C) were used as the reference standard.

## Materials and methods

### Patients

RA patients were prospectively recruited at the Rheumatology Clinic from 2022 to 2024. All patients fulfilled the 2010 American College of Rheumatology (ACR)/European League Against Rheumatism classification (EULAR) criteria and were regularly followed up clinically. MRI examination was performed within 4 weeks of clinical assessment. Patients who refused MRI examination or had a contraindication to MRI were excluded. The study protocol was approved by the Clinical Research Ethics Committee of the Joint Chinese University of Hong Kong (CUHK) and the New Territories East Cluster (NTEC) (Ref. No: 2021.398), with signed informed consent obtained from all participants.

### Clinical assessment

Clinical assessments were performed at the Rheumatology Clinic and included the Disease Activity Score 28 (DAS28) and Simple Disease Activity Index (SDAI) to assess disease activity, as well as Health Assessment Questionnaire–Disability Index (HAQ-DI), to assess functional status. Serology (ESR, CRP) was also performed.

### MRI protocol

MRI examination of the most clinically affected wrist was performed, in the prone position, on a 3.0-T system (Philips Ingenia, Best, Netherlands). Scan plane extended from the distal radio-ulnar to the carpometacarpal joints, using a dedicated wrist coil. The scan protocol was designed to assess inflammatory and structural RA-related changes (Table 1).

**Table 1:** MRI examination parameters for RA wrist assessment

Sequence	TR (ms)	TE (ms)	Flip angle	Bandwidth (Hz per pixel)	Matrix size (mm)	FOV (mm x mm)	NEX	Slice thickness (mm)	Slice gap (mm)	Acquisition time
T1WI_TRA	591	12	90°	157	268 × 216	80 × 80	2.5	3	0.3	2 min 20 s
T1WI_FS_TRA	735	12	90°	157	268 × 216	80 × 80	2	3	0.3	2 min 52 s
T2WI_FS_TRA	3273	80	90°	132.7	200 × 143	80 × 80	2	3	0.3	2 min
PDWI_TRA	3045	25	90°	238.9	320 × 238	80 × 80	2	2.5	0.5	2 min 26 s
PDWI_FS_TRA	3616	25	90°	222.6	320 × 238	80 × 80	2	2.5	0.5	4 min
T1WI_FS_TRA + C	735	12	90°	157	268 × 216	80 × 80	2	3	0.3	2 min 52 s
T2FS_COR	3117	70	90°	217.6	228 × 180	80 × 80	1	1.5	0.15	2 min 10 s
T1W_COR	582	12	90°	142	200 × 160	80 × 80	1	1.5	0.15	1 min 55 s

*TR* repetition time, *TE* echo time, *TI* inversion time, *FOV* field of view, *NEX* number of excitations

### MRI analysis

All MRI measurements were made at a personal computer using dedicated DICOM image assessment software (RadiAnt). Synovitis and tenosynovitis were scored using the RAMRIS system [12]. Two radiologists (with 3 and 4 years of MRI reporting experience, respectively, much of which focused on assessment of inflammatory and structural wrist MRI changes) independently compared synovitis and tenosynovitis severity on the following three sequences: T2FS, PDFS, and T1FS + C. The more experienced (4 years) reader was designated as the main reader. Both readers were trained by a senior musculoskeletal radiologist with more than 30 years of MRI reporting experience. Both readers independently scored each sequence group. Only after all scoring was completed were the results compared and analyzed. Both readers repeated the same scoring 2 weeks after the initial assessment, blinded to the earlier assessments. The scoring order was randomized during assessment.

#### Synovitis grading

Synovitis was evaluated on axial images. The wrist joint was divided into three areas, namely the radio-ulnar joint, the radio-carpal joints, and the intercarpal and carpometacarpal joints. Synovitis was graded from 0 to 3. Grade 0: no synovitis. Grade 1: mild synovitis, i.e., less than 1/3 of the expected maximum synovial volume or thickness. Grade 2: moderate synovitis, i.e., 1/3 to 2/3 of the expected maximum synovial volume or thickness. Grade 3: severe synovitis, i.e., close to or at the expected maximum synovial volume or thickness.

#### Tenosynovitis grading

Tenosynovitis was evaluated on axial images at nine areas: the six extensor tendons and three flexor tendon compartments. Each tendon compartment was scored separately from 0 to 3. Grade 0: no tendon sheath effusion or post-contrast enhancement. Grade 1: < 1.5 mm tendon sheath effusion ± post-contrast enhancement. Grade 2: ≥ 1.5 and < 3.0 mm tendon sheath effusion ± post-contrast enhancement. Grade 3: ≥ 3.0 mm tendon sheath effusion ± post-contrast enhancement.

### Statistical analysis

SPSS software (SPSS for Microsoft Windows, version 24.0, IBM) was used. Quantitative variables were expressed as means, standard deviations (SD), and ranges. Qualitative variables were expressed as raw numbers, proportions, and percentages. Inter-sequence agreement, inter-observer and intra-observer agreement were assessed using two-way mixed effects, consistency, single rater/measurement intraclass Pearson correlation coefficients (ICC) with 95% confidence intervals (95% CI). The strength of agreement was interpreted as follows: ≤ 0, poor; 0.01–0.20, slight; 0.21–0.40, fair; 0.41–0.60, moderate; 0.61–0.80, substantial; and ≥ 0.81, excellent. Bland–Altman plots were constructed for the inter-sequence comparison from which the mean bias (indicating a potential overestimation from one reader compared to another one), the regression line of paired differences (indicating a potential proportionality between differences and the magnitude of measurements), and the limits of agreement (defined as mean bias 1.96 SD on paired differences) were derived.

## Results

### Patients

Seventy-seven RA patients (age 61 ± 12 years), of whom 64 (83%) were female, were studied. Demographic and clinical characteristics are presented in Table 2. Average disease duration was 8.2 years.

**Table 2:** Demographic and clinical characteristics of RA patients

Patient characteristic	*n* = 77
Age (years)	61.0 ± 12.2
Female sex (%)	64 (83.1)
Pain score	2.4 ± 2.4
Patient global assessment	2.7 ± 2.2
Physician global assessment	1.7 ± 2.0
DAS28 index	2.5 ± 1.0
DAS28 remission* (%)	51 (66.2)
SDAI index	7.7 ± 7.5
SDAI remission** (%)	32 (41.6)
ESR	34.6 ± 24.1
CRP	5.9 ± 12.4
HAQ	0.4 ± 0.4

*DAS* disease activity score, *SDAI* simple disease activity index, *BME* bone marrow edema, *CRP* C-reactive protein, *HAQ* Health Assessment Questionnaire

* Defined as DAS28 < 2.6

** Defined as SDAI ≤ 3.3

#### Synovitis grading

Reliability results are shown in Table 3. For grading synovitis severity, interclass correlation between readers was excellent for all sequences (ICC: 0.929–0.948). Intraclass correlation for scoring synovitis severity was also excellent for both readers (0.927–0.974).

**Table 3:** Reliability of both readers for scoring synovitis and tenosynovitis severity on T2FS, PDFS, and T1FS + C sequences (*n* = 77)

	Synovitis score	Tenosynovitis score
Interclass correlation coefficient		
T2FS	0.933	0.872
PDFS	0.929	0.876
T1FS + C	0.948	0.924
Intraclass correlation coefficient		
Reader 1		
T2FS	0.927	0.854
PDFS	0.966	0.925
T1FS + C	0.974	0.849
Reader 2		
T2FS	0.962	0.804
PDFS	0.949	0.831
T1FS + C	0.958	0.902

**Fig. 1:**
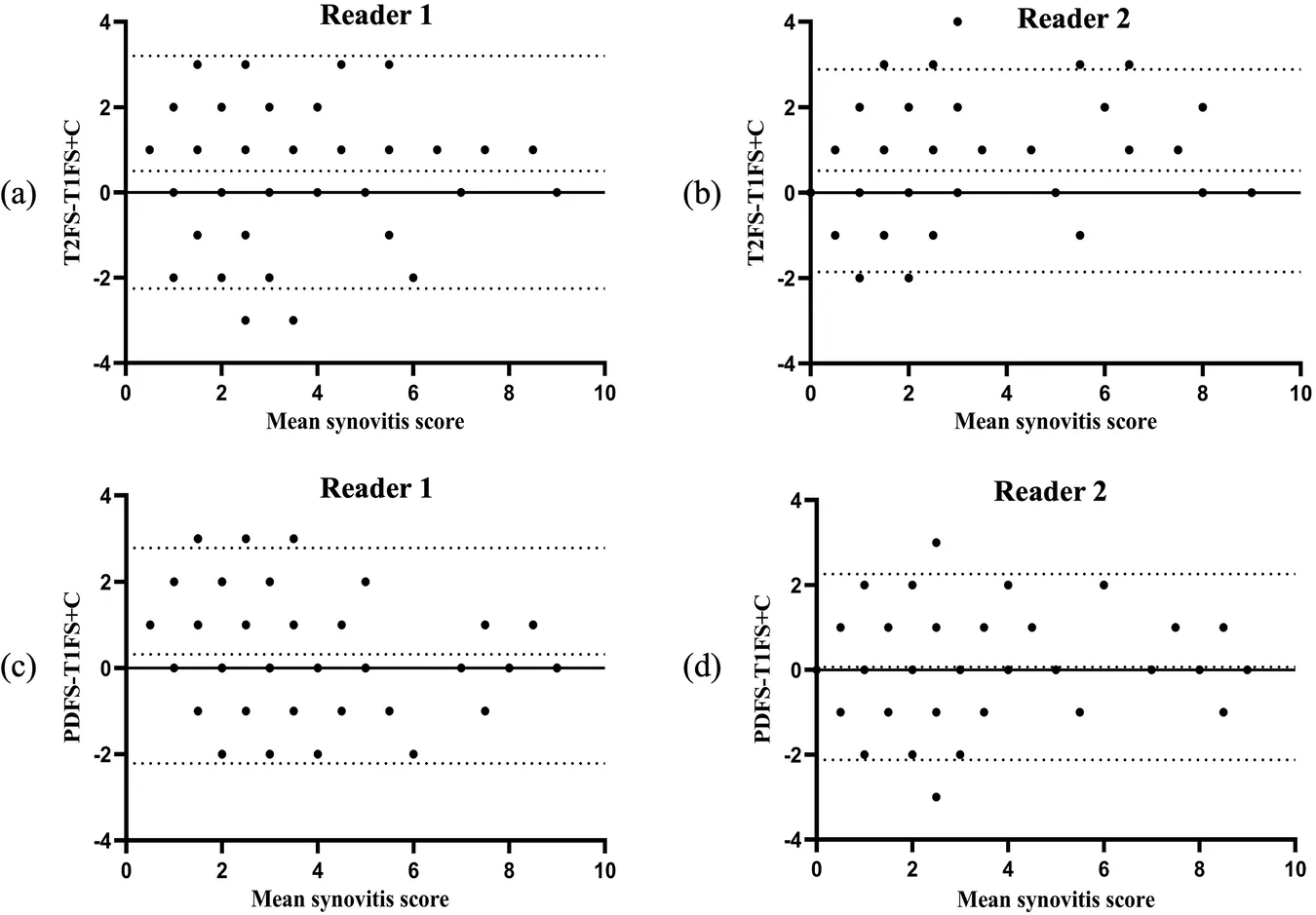
Bland–Altman plots of synovitis RAMRIS severity scores on T2FS and PDFS readers compared to T1FS + C imaging for readers 1 and reader 2. The difference between paired T2FS and PDFS measurements compared to T1FS + C imaging is plotted against the means of the two measures. The solid zero-baseline line represents no difference between both (T2FS and PDFS) measurements when compared to T1FS + C imaging. The dashed central line is the average of the differences between all T2FS and PDFS measurements compared to T1FS + C imaging for that reader. A central line located close to zero baseline indicates small differences between synovitis severity scores for both sequences. A middle line located above zero indicates that both readers tend to marginally overgrade synovitis on T2FS and PDFS sequences compared to T1FS + C imaging. Each dot on the graph may represent more than one patient when the differences for both patients is similar. Both readers showed better agreement with T1FS + C imaging for PDFS than with T2FS imaging for synovitis scoring. **a** T2FS imaging for reader 1. **b** T2FS imaging for reader 2. **c** PDFS imaging for reader 1. **d** PDFS imaging for reader 2

Bland–Altman plot analysis showed that PDFS synovitis severity scores were more similar to T1FS + C synovitis scores than T2FS synovitis scores for both readers (Fig. 1). Average synovitis score for T2FS imaging was higher than that for T1FS + C and PDFS imaging (Table 4). For synovitis severity, PDFS severity scoring was almost identical to T1FS + C scoring, while T2FS scoring overestimated synovitis severity by up to 19% (Table 4). Similar patterns were seen when different wrist subregions were scored (Table 5). The correlation between PDFS and T2FS with T1FS + C imaging for both readers is shown in Table 4.

**Table 4:** RAMRIS synovitis and tenosynovitis severity score and correlation of T2FS, PDFS with T1FS + C sequences for both readers

	Synovitis score	ICC	Tenosynovitis score	ICC
Reader 1				
T1FS + C	3.2 ± 2.6	——	2.8 ± 2.5	——
T2FS	3.8 ± 2.4	0.897	4.3 ± 2.6	0.731
PDFS	3.2 ± 2.3	0.931	3.9 ± 2.5	0.856
Reader 2				
T1FS + C	3.1 ± 2.7	——	3.6 ± 2.6	——
T2FS	3.6 ± 2.7	0.948	4.4 ± 2.7	0.800
PDFS	3.1 ± 2.7	0.969	4.5 ± 2.3	0.808

**Table 5:** Synovitis and tenosynovitis scoring of anatomical subregions of two readers for different sequences

	Reader 1			Reader 2		
	T2FS	PDFS	T1FS + C	T2FS	PDFS	T1FS + C
Synovitis score						
Radio-ulnar joints	1.3 ± 1.1	1.1 ± 1.2	1.1 ± 1.2	1.1 ± 1.1	1.1 ± 1.0	1.1 ± 1.1
Radio-carpal joint	1.7 ± 0.8	1.2 ± 0.8	1.6 ± 0.8	1.8 ± 0.9	1.1 ± 0.9	1.2 ± 1.0
CMC joint	0.9 ± 1.0	0.9 ± 0.9	0.7 ± 0.9	0.7 ± 1.1	0.8 ± 1.1	0.8 ± 1.1
Synovitis total score	3.8 ± 2.4	3.2 ± 2.3	3.2 ± 2.6	3.6 ± 2.7	3.1 ± 2.7	3.1 ± 2.7
Tenosynovitis score						
Extensor tendon (I–VI)	2.8 ± 2.0	2.2 ± 1.9	1.5 ± 1.5	2.8 ± 2.0	3.0 ± 1.8	2.2 ± 1.8
FCR	0.5 ± 1.0	0.5 ± 0.7	0.5 ± 0.7	0.6 ± 0.8	0.5 ± 0.9	0.7 ± 0.9
FPL	0.5 ± 0.6	0.5 ± 0.8	0.2 ± 0.3	0.5 ± 0.7	0.5 ± 0.7	0.2 ± 0.6
Ulnar bursa	0.5 ± 0.8	0.6 ± 0.8	0.5 ± 0.5	0.5 ± 0.7	0.5 ± 0.7	0.5 ± 0.8
Tenosynovitis total score	4.3 ± 2.6	3.9 ± 2.5	2.8 ± 2.5	4.4 ± 2.7	4.5 ± 2.3	3.6 ± 2.6

#### Tenosynovitis grading

Reliability results are shown in Table 3. For grading tenosynovitis severity, interclass correlation between reader 1 and reader 2 was good to excellent for all sequences (ICC: 0.872–0.924) (Table 3). Intraclass correlation for scoring tenosynovitis severity was also good to excellent agreement for both readers (0.804–0.925) (Table 3).

**Fig. 2:**
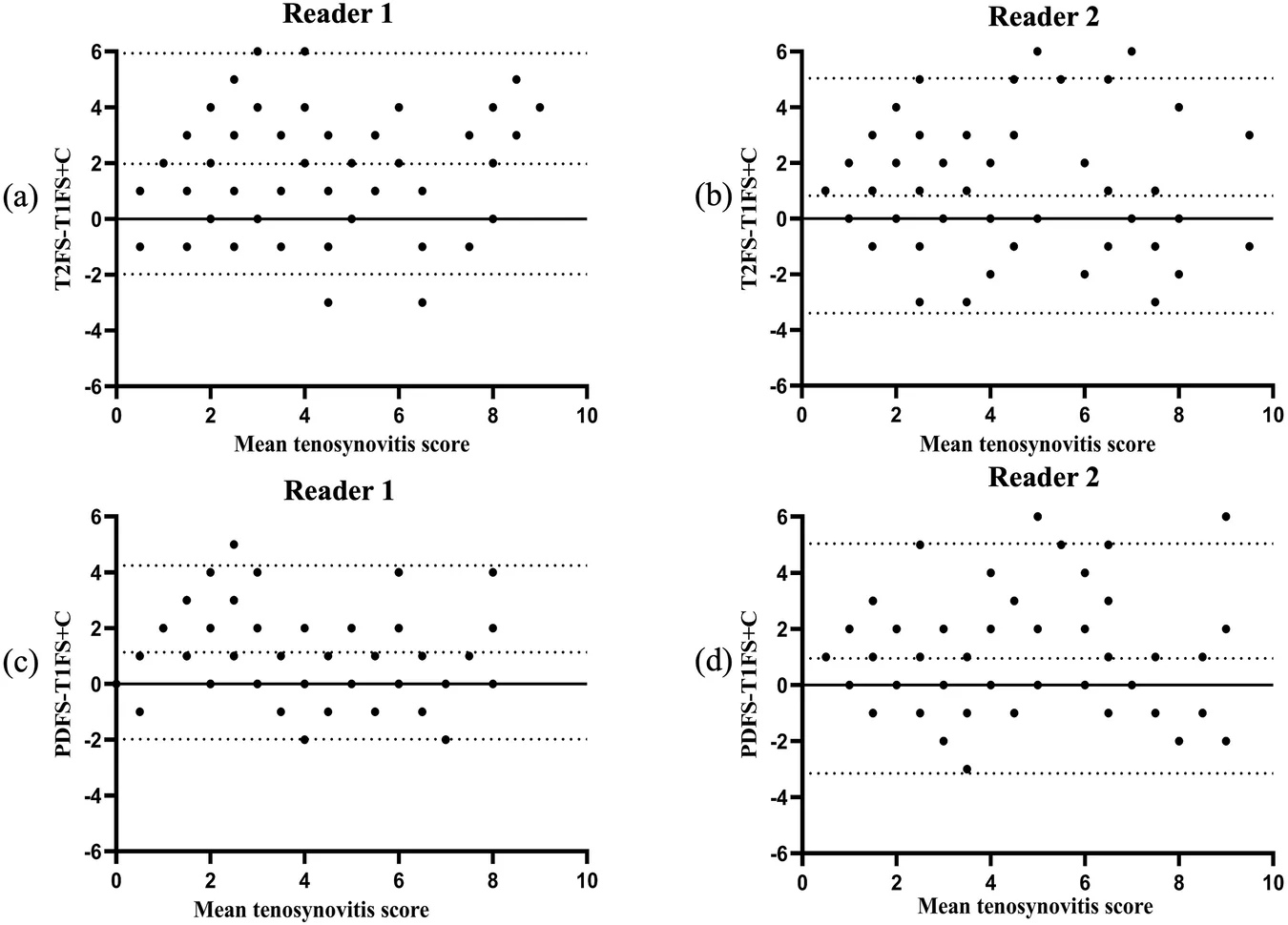
Bland–Altman plots of tenosynovitis RAMRIS severity scores on T2FS and PDFS readers compared to T1FS + C imaging for readers 1 and reader 2. The difference between paired T2FS and PDFS measurements compared to T1FS + C imaging is plotted against the means of the two measures. The solid zero-baseline line represents no difference between both (T2FS and PDFS) measurements when compared to T1FS + C imaging. The dashed central line is the average of the differences between all T2FS and PDFS measurements compared to T1FS + C imaging for that reader. A dashed central line located close to the zero baseline indicates small differences between tenosynovitis severity scores for both sequences. A middle line located above zero indicates that both readers tend to overgrade tenosynovitis on T2FS and PDFS sequences compared to T1FS + C imaging. Each dot on the graph may represent more than one patient when the differences for both patients are similar. **a** T2FS imaging for reader 1. **b** T2FS imaging for reader 2. **c** PDFS imaging for reader 1. **d** PDFS imaging for reader 2

Bland–Altman plot analysis showed that PDFS tenosynovitis severity scores were more similar to T1FS + C tenosynovitis severity scores than T2FS tenosynovitis severity scores for both readers (Fig. 2). Average tenosynovitis severity scores were higher for PDFS (*p* < 0.001) and T2FS (*p* < 0.001) images than T1FS + C imaging (Table 4). For tenosynovitis severity, PDFS and T2FS both yielded higher tenosynovitis severity scores than T1FS + C (*p* < 0.001) (Table 4). Compared to T1FS + C imaging, PDFS tended to overestimate tenosynovitis severity by up to 39%, while T2FS tended to overestimate tenosynovitis severity by up to 54% (Table 4). Similar patterns were seen when different wrist subregions were scored (Table 5). The correlation between PDFS and T2FS with T1FS + C imaging for both readers is shown in Table 4.

## Discussion

MRI identifies inflammatory changes and structural damage within and around joints [4, 16]. MRI can be utilized to help diagnose inflammatory arthropathy, quantify the type and level of inflammatory activity and structural damage, and determine response to treatment [16]. The degree of structural damage evident on MRI at presentation is a better predictor of long-term structural damage progression than clinical, radiographic, or serological features [17]. The main limitations to more frequent usage of MRI are its cost and limited availability, which are partially related to long scan times, exacerbated by the perceived need for intravenous contrast administration [18]. The main purpose of contrast administration in RA patients is to identify enhancing (i.e., hyperperfused) synovial or tenosynovial tissue [16]. In this study, we investigated whether two non-contrast sequences, namely T2FS and PDFS, could accurately quantify the level of synovial or tenosynovial inflammation, using T1FS + C imaging as the comparative standard.

**Fig. 3:**
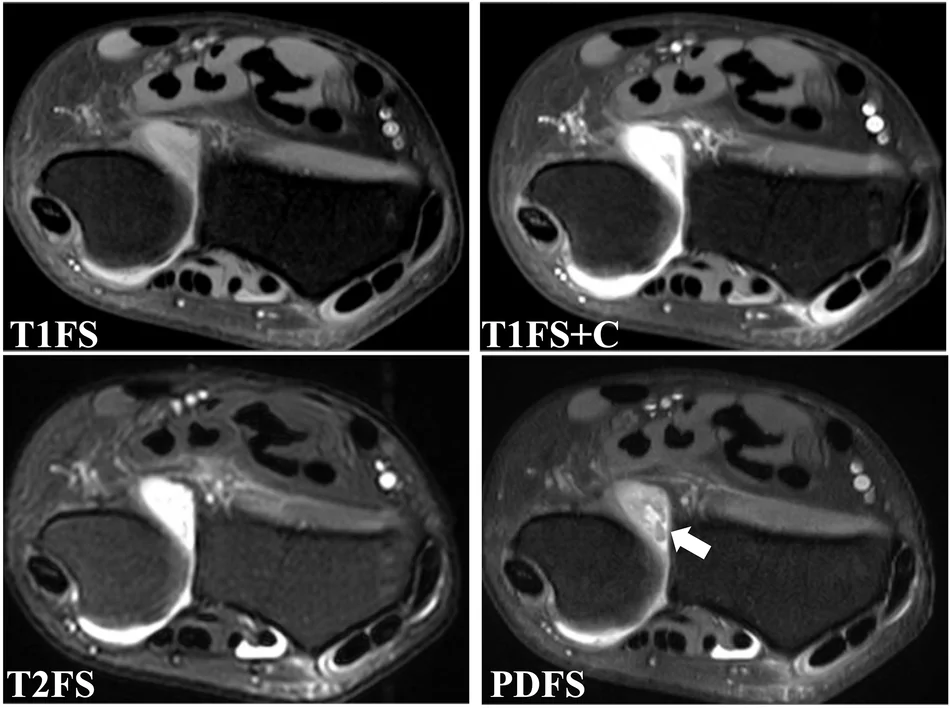
31-year-old female patient with severe synovitis of the radio-ulnar joint seen on different sequences. The thickened synovium is better seen on PDFS imaging than T1FS + C and T2FS imaging (arrow). In this patient, the radio-ulnar joint synovitis is hyperintense, which most likely indicates highly proteinaceous synovial proliferation on the T1FS sequence before contrast, which indicates severe synovial pathology

**Fig. 4:**
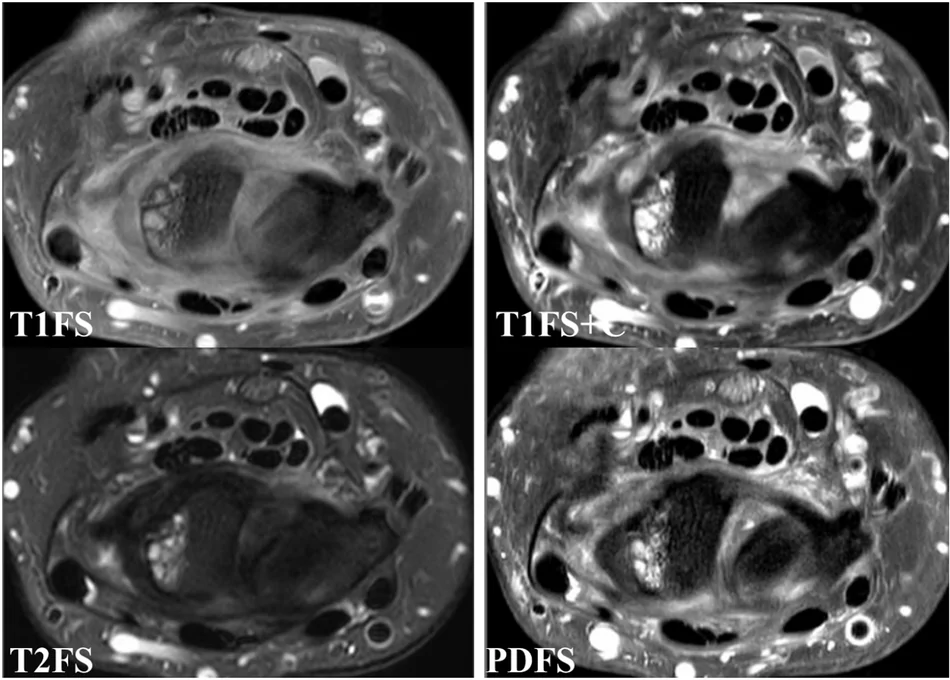
60-year-old female with moderate severity radio-carpal joint synovitis seen on different sequences. Synovitis, as shown by thickening of synovium, is more conspicuous on PDFS imaging than T2FS imaging and is also more comparable to T1FS + C imaging than T2FS imaging (arrow)

This study showed that PDFS sequences performed better than T2FS sequences for grading both synovitis and tenosynovitis severity. For grading synovitis severity, PDFS imaging had RAMRIS severity scores almost identical to T1FS + C imaging, as well as excellent agreement with T1FS + C imaging and excellent intra- and interclass correlation (Fig. 3). T2FS imaging tended to overestimate synovitis and had poorer intra- and interclass correlation for both readers than PDFS imaging (Fig. 4). The most likely reason for better quantification of synovitis severity with PDFS rather than T2FS imaging is that PDFS imaging reflects differences in proton content between tissues and thus provides better tissue contrast and visualization of non-fatty soft tissues compared to T2FS imaging, which is primarily geared toward being fluid sensitive [19, 20]. This leads to synovitis being more conspicuous on PDFS than T2FS imaging (Fig. 4). Also, the T1FS + C sequence utilized had a relatively long TR to mitigate T1-weighting of native tissues and thereby maximized gadolinium-induced T1-shortening as the primary source of contrast. These T1FS + C sequence parameters are standard [18, 21] and can result in post-contrast imaging contrast resembling that of PDFS.

**Fig. 5:**
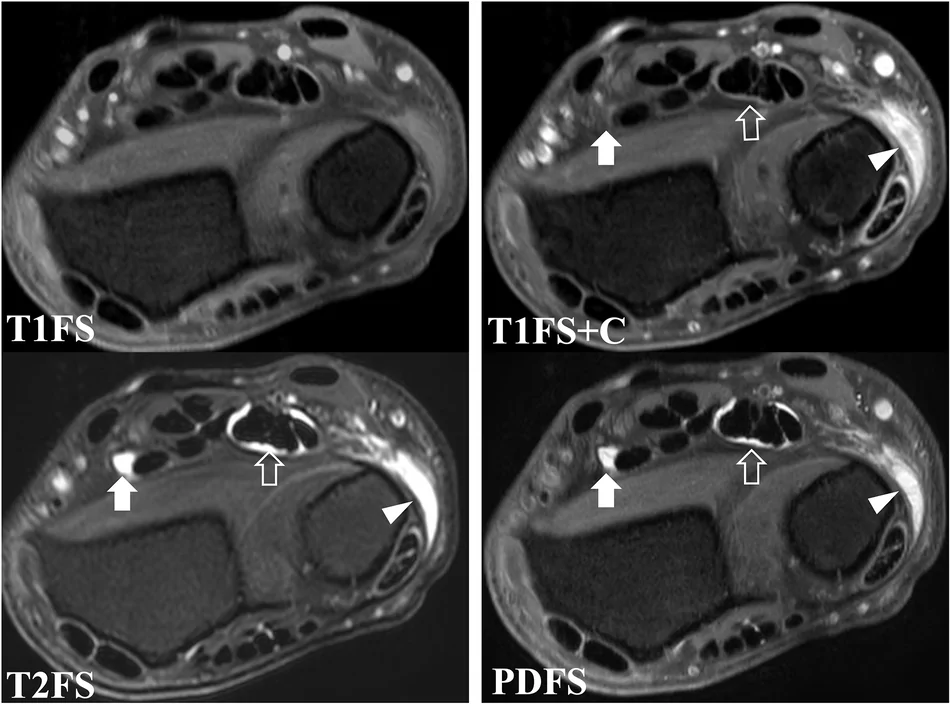
78-year-old female patient with moderate tendon sheath effusions of the flexor pollicis longus tendon sheath (arrow) and ulnar bursa (open arrow) flexor tendon compartment. Tendon sheath effusion is more conspicuous on both T2FS and PDFS imaging compared to T1FS + C imaging. The synovial proliferation (arrowheads) evident on the ulnar aspect of the wrist on PDFS imaging looks more similar to T1FS + C imaging than T2FS imaging

For grading tenosynovitis, PDFS and T2FS imaging consistently yielded higher tenosynovitis severity scores compared to T1FS + C imaging. This higher tenosynovitis RAMRIS scoring with non-contrast PDFS and T2FS imaging is most likely due to tendon sheath effusions resulting in hyperintense tendon sheath distension on PDFS and T2FS imaging, which does not usually enhance avidly with contrast. This leads to increased conspicuity of tenosynovitis on non-contrast, rather than post-contrast sequences. T1FS + C imaging highlights enhancing tenosynovium but does not highlight the tendon sheath effusion, which is seen as a non-enhancing isointense rim around tendons (Fig. 5). As tendon sheath effusion is a marker of tenosynovitis, it could be argued that T1FS + C imaging possibly underestimates tenosynovitis severity while both PDFS and T2FS imaging provide a truer measure of tenosynovitis. That said, RAMRIS scoring is based on T1FS + C imaging, and this was used as the assessment method in this study. The RAMRIS scoring system is a semi-quantitative, observer-dependent tool developed and validated for use in clinical trials, but not necessarily applicable in daily practice. For clinicians interpreting RAMRIS scores, our data suggest a need for cautious interpretation as PDFS/T2FS will yield a higher tenosynovitis severity score than T1FS + C imaging. This does not invalidate the utility of non-contrast MRI for monitoring wrist tenosynovitis in RA patients, but it does imply that the threshold for defining inflammatory activity or change should be considered sequence-specific. PDFS also had a higher correlation than T2FS imaging compared to T1FS + C imaging for tenosynovitis scoring, though the correlation was not as high as that seen with synovitis. PDFS scan time (4 min) was longer than T1FS + C (2 min 52 s), but post-contrast imaging also involves preliminary plain sequence scanning, contrast agent injection, and additional observation regarding the possibility of allergic reaction. As such, the total time needed for T1FS + C is much longer than PDFS.

Several previous studies have addressed the possibility of using various non-contrast sequences as an alternative to T1FS + C imaging to quantify wrist synovitis and tenosynovitis severity in RA patients [18–23]. No previous studies have assessed the value of PDFS imaging in this regard. As found in our study, T2FS imaging was found to correlate well with T1FS + C imaging for assessing synovitis [21, 22] with correlation coefficients ranging from 0.75 to 0.82 [22] and with good reader reliability [21, 22]. Also, as shown in previous studies, tenosynovitis tended to have higher RAMRIS scores on T2FS imaging than T1FS + C imaging, as tendon sheath effusions are more conspicuous on fluid-sensitive sequences [18, 21, 22]. The Dixon technique was found to correlate poorly (r = 0.36) with T1FS + C imaging for quantifying synovitis, though it had a modest (r = 0.64) correlation for revealing tenosynovitis [20, 22]. In contrast, excellent correlation was found between double inversion recovery (DIR) imaging, which enables both high contrast and high spatial resolution, and T1FS + C imaging for assessing both the presence and severity of wrist synovitis [23]. This DIR-based study, however, only employed coronal plane imaging, which is sub-optimal to axial imaging for quantifying wrist synovitis and tenosynovitis [22, 23].

The main limitation of our study is that the assessment was confined to three MRI sequences only, and other sequences, such as DIR imaging, Dixon, or 3D Dixon sequences, were not included. Second, this study was conducted exclusively on the wrist joint, which limits the applicability of findings to other commonly involved joints, such as the metacarpophalangeal (MCP), metatarsophalangeal (MTP), or knee joints. In this study, only synovitis and tenosynovitis severity were compared between different sequences. Further study should be performed to find out if PDFS also showed good performance in other RA-related parameters and joints. Third, as only RA patients were studied, the value of non-contrast sequences in other arthritic conditions, such as psoriatic arthritis or osteoarthritis, was not assessed. Fourth, the limited MR experience of the two readers (at 3 and 4 years) may hinder the generalizability of results, though both readers had considerable experience in evaluating MRI wrist images and had excellent reliability for evaluating inflammatory severity changes. Finally, as most patients in the study had mild to moderate synovitis, the ability of T2FS and PDFS imaging for detecting subtle synovitis compared to post-contrast imaging was not fully addressed.

In conclusion, this study shows that PDFS imaging provides comparable semi-quantitative scoring to T1FS + C when evaluating wrist synovitis with good to excellent intra- and inter-reader reliability for quantifying both wrist synovitis and tenosynovitis severity in RA patients. Tenosynovitis severity tends to be overestimated on PDFS and T2FS imaging compared to T1FS + C imaging, due to increased conspicuity of tendon sheath effusions. PDFS imaging can be used as an alternative to contrast imaging in the assessment of wrist synovitis in RA patients.

## Data Availability

The data underlying this article will be shared on reasonable request to the corresponding author.

## Abbreviations

ICC: Interclass correlation coefficient
PDFS: Proton-density imaging with fat-saturation
RAMRIS: Rheumatoid arthritis magnetic resonance imaging score
RA: Rheumatoid arthritis
T1FS + C: Post-contrast T1-fat-saturated
